# Cathodic electrolyte engineering toward durable Zn–Mn aqueous batteries

**DOI:** 10.1093/nsr/nwad265

**Published:** 2023-10-11

**Authors:** Wanhai Zhou, Hong Jin Fan, Dongyuan Zhao, Dongliang Chao

**Affiliations:** Laboratory of Advanced Materials, Shanghai Key Laboratory of Molecular Catalysis and Innovative Materials, State Key Laboratory of Molecular Engineering of Polymers, College of Chemistry and Materials, Fudan University, China and; School of Physical and Mathematical Sciences, Nanyang Technological University, Singapore; Laboratory of Advanced Materials, Shanghai Key Laboratory of Molecular Catalysis and Innovative Materials, State Key Laboratory of Molecular Engineering of Polymers, College of Chemistry and Materials, Fudan University, China and; Laboratory of Advanced Materials, Shanghai Key Laboratory of Molecular Catalysis and Innovative Materials, State Key Laboratory of Molecular Engineering of Polymers, College of Chemistry and Materials, Fudan University, China and

Zn–Mn aqueous batteries (ZMABs) present potential for grid-scale energy storage with the benefits of low cost, high safety and eco-friendliness [1]. Since 1866 (Leclanché wet cell), we have witnessed the prosperity of ZMABs in the primary battery market and an increasing interest in rechargeable ZMABs. In the last 5 years, achievements have been made in high-capacity MnO_2_ cathodes, dendrite-free Zn metal anodes and functionalized electrolytes [2–4], which push rechargeable ZMABs a step closer to practical applications. In particular, electrolyte regulation has been regarded as most important in stabilizing the interface but it remains challenging. Recently, writing in *National Science Review*, Liang, Fang and co-workers reported a lean-water quasi-eutectic electrolyte (QEE) that has been shown to be beneficial in facilitating the reversible interfacial deposition and reaction kinetic of Mn-based cathodes in a long cycle process [5].

The development of rechargeable ZMABs is still in the primary stage. The competitiveness of ZMABs is mainly beset by their unsatisfied energy density and poor lifespan. The energy density is affected by two aspects: the specific capacity and the output voltage. The capacity of the MnO_2_ cathode is dependent on the electrolyte. For instance, the pH of the electrolyte determines the working mechanism of a MnO_2_ cathode with 1 or 2 e^–^ transfer. Considering the narrow electrochemical stability window (ESW) of water (∼1.23 V), electrolytes may directly limit the output voltage. Recent reports have shown that regulating pH and using water-in-salt electrolytes are effective in widening the ESW [1,6], which can endow ZMABs with a high voltage of >2 V. As for the lifespan, the instability of the MnO_2_ cathode can be caused by three main reasons (illustrated in Fig. 1a): the irreversible MnO_2_ → Mn^2+^ dissolution, the passivation of the formed zinc hydroxide sulfate (Zn_4_(SO_4_)(OH)6·*n*H_2_O, ZHS) byproduct and the accumulation of dead MnO_2_ [2,3,7]. Importantly, these failure processes are closely related to the electrolyte. For instance, the dead MnO_2_ stems from the increase in pH and the generation of ZHS needs to consume ions in the electrolyte including Zn^2+^, SO_4_^2–^ and OH^–^. Therefore, electrolyte optimization is of pivotal importance. Typical mitigation strategies include pH balance, solvation regulation and redox mediation for durable ZMABs, which are to be elaborated as follows.

**Figure 1.**
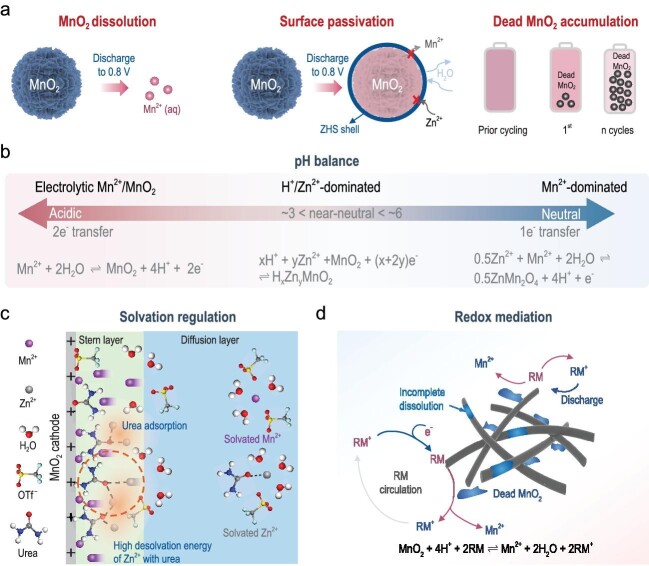
Summary of challenges and electrolyte engineering of MnO_2_ cathodes toward durable Zn–MnO_2_ aqueous batteries. (a) Illustration of the three major failure mechanisms of the MnO_2_ cathode. (b) Summary of pH regulation for changing the reaction mechanism of the MnO_2_ cathode. Reprinted with permission from [2]. (c) Diagram of solvation structure regulation by using a urea-based eutectic electrolyte at the cathode interface. Reprinted with permission from [5]. (d) Schematic illustration of the elimination of dead MnO_2_ using a redox mediator (RM).

## pH BALANCE

The current consensus of energy-storage mechanisms in ZMABs focuses on H^+^ and/or Zn^2+^-dominated insertion/deinsertion and Mn^2+^-dominated dissolution/deposition reactions that are associated with a pH clue (proton-coupled reaction) [2]. As summarized in Fig. 1b, in mild pH (3–6) electrolytes, typically in 2 M ZnSO_4_ electrolyte, the Mn^2+^ deposition/dissolution (usually 1 e^–^ transfer) would mix with Zn^2+^ deinsertion/insertion and the corresponding dominant reaction changes with the variation in pH values, causing abnormal capacity fluctuations. The possible factors that cause the pH increase include an irreversible H^+^ de-intercalation reaction and MnOOH disproportionation reaction in the cathode, hydrogen evolution reaction and corrosion, side reactions of the Zn anode and a backward hydrolysis reaction due to Zn^2+^/Mn^2+^ consumption after cycles [2]. The appearance of ZHS can work as a buffer layer to stabilize the pH. However, ZHS is poor in both electrical and ionic conductivity, greatly restraining the charge transport [7]. As a result, introducing pH buffer electrolytes or additives, such as H_2_PO_4_^–^ and CH3COO^–^, can act as a proton buffer reservoir to maintain the pH value toward durable ZMABs [2]. In addition, further decreasing the pH to acid (pH < 3, e.g. ZnSO_4_ + MnSO_4_ + 0.1 M H_2_SO_4_) can facilitate the favorable two-electron electrolytic Mn^2+^ ↔ MnO_2_ reaction, which provides a high capacity of ≈600 mAh g^−1^ and also a high voltage plateau of 2 V [6].

## SOLVATION REGULATION

To suppress the MnO_2_ dissolution caused by the Jahn–Teller effect, Mn^2+^ additives into the electrolyte have been applied in almost every ZMAB. Differently from the extensive attention on the Zn^2+^ solvation structure, reports related to the Mn^2+^ solvation structure are relatively fewer. This is partly due to the lack of awareness and partly because of the challenges of detection using conventional tests such as Raman spectra with the small quantities of manganese ions in the electrolyte. As proposed by Chen and Co-workers [8], the cationic accelerator (CA), such as poly(vinylpyrrolidone) (PVP), would change the hydrated ion structures of [Mn(H_2_O)_6_]^2+^ to CA–[Mn(H_2_O)_5_]^2+^. It is shown that the CA[Mn(H_2_O)_5_]^2+^ can carry cations to migrate to the electrode surface and undergo a rapid desolvation process. This effectively accelerates the electrolytic reaction of the MnO_2_ cathode and benefits from a long lifespan of 2000 cycles. In addition, the solvation structure regulation can optimize the solid–liquid interfacial state that dominates the Mn dissolution/deposition process. As shown in Fig. 1c, in QEE, owing to the strong hydrogen bond interaction between the C−F group (in OTf^−^) and the −NH_2_ group (in urea), the substitution of OTf^−^ by urea converts the cathode interface from anion enrichment to molecular enrichment [5]. The high binding force between Zn^2+^ and urea makes the desolvation of Zn^2+^ at the cathode interface more difficult than Mn^2+^, thus restraining the mass transfer of Zn^2+^, which reduces the Zn^2+^ deposition at the interface while improving the reversibility of the Mn^2+^ deposition. As a consequence, such a mass transfer modulation by QEE increases the valence of the Mn and decreases the content of Zn in the deposition, contributing to high capacity and reaction reversibility. Meanwhile, according to the authors, the enrichment of the molecular interface raises the Stern layer potential and the repulsive force *VR* at the cathode interface. A higher Stern layer potential, although detrimental to the initial cathodic kinetics, avoids the agglomeration of deposits and thus optimizes the cathodic kinetics during the stabilization cycle for durable ZMABs.

## REDOX MEDIATION

The abnormal attenuation of the cathode can be triggered by the inert Zn/Mn oxides and devitalized MnO_2_ aggregation (‘dead Mn’) [2], especially in high-areal-capacity redox-flow ZMABs [9]. So far, the redox mediator (RM) strategy, offering an additional charge-transfer route beyond the localized interface, is an effective in eliminating dead Mn, simultaneously enabling sufficient, homogeneous and fast redox reaction of the Mn-based cathode [10]. As a discharge RM (Fig. 1d), the redox potential needs to be lower than that for Mn^2+^/MnO_2_. Thus, it can be utilized to spontaneously reduce solid MnO_2_ during discharging based on Equation (1):


(1) \begin{eqnarray*}
&& {\mathrm{Mn}}{{\mathrm{O}}}_2 + 4{{\mathrm{H}}}^ + + 2{\mathrm{RM}} \leftrightarrow 2{{\mathrm{H}}}_2{\mathrm{O}}\nonumber\\
&&\quad + {\mathrm{M}}{{\mathrm{n}}}^{2 + } + 2{\mathrm{R}}{{\mathrm{M}}}^ + ,\Delta {\mathrm{G}} < 0.
\end{eqnarray*}

Proposed by Lu and co-workers, the I^–^/I_3_^–^-mediated neutral ZMAB using an acetate electrolyte can achieve a high areal capacity of ∼50 mAh cm^–2^ for >50 cycles [9]. Despite its beneficial role in boosting the performance of redox-flow ZMABs, the metrics of the RM design also need to be evaluated regarding its solubility, electrochemical reversibility, kinetics and stability.

## OUTLOOK

To conclude, electrolyte optimizations via pH balance, solvation regulation and redox mediation are available and effective for addressing the challenges of dissolution, ZHS passivation and dead Mn in MnO_2_ cathodes for durable ZMABs. Looking into future, we propose the following research directions:

- Design novel pH buffer electrolytes or additives that can maintain stable pH and restrain the passivation of the cathode.
- Regulate a Mn^2+^ solvation structure that accelerates and stabilizes the deposition/dissolution reaction of the MnO_2_ cathode without dead Mn.
- Develop stable redox mediators with a low crossover that can facilitate reversible reactions of the MnO_2_ cathode with a small potential gap.
- Develop wide-temperature electrolytes that endow durable ZMABs with excellent low- and high-temperature performances.
